# Childhood trauma in bipolar disorder: new targets for future interventions

**DOI:** 10.1192/bjo.2022.529

**Published:** 2022-07-11

**Authors:** Danielle Hett, Bruno Etain, Steven Marwaha

**Affiliations:** Institute for Mental Health, University of Birmingham, UK; and National Centre for Mental Health, The Barberry, Birmingham and Solihull Mental Health Trust, UK; INSERM UMR-S 1144, Université de Paris, France; Institute for Mental Health, University of Birmingham, UK; National Centre for Mental Health, The Barberry, Birmingham and Solihull Mental Health Trust, UK; and Specialist Mood Disorders Clinic, Zinnia Centre, Birmingham and Solihull Mental Health Trust, UK

**Keywords:** Bipolar affective disorders, trauma, childhood trauma, early intervention, affective instability

## Abstract

Childhood trauma, particularly emotional abuse, is prevalent in bipolar disorder, and affective instability mechanistically explains the relationship between childhood trauma and poor bipolar disorder outcomes. Yet, trauma-focused interventions in bipolar disorder are lacking. This editorial calls for future early interventions to target the effects of childhood trauma and affective instability in this population.

Bipolar disorder is a debilitating, multi-system mental illness marked by fluctuations in activity, cognition and mood. It affects approximately 1% of the general population^1,2^ and is associated with an increased risk of suicide, worsened functioning, poor quality of life, cognitive impairment and physical morbidity. Because of its recurrent, and in some people, progressive nature, bipolar disorder is a prime candidate for early intervention. There is little early intervention clinical activity in bipolar disorder, and the effectiveness of early intervention strategies remain unclear. In this paper, we propose one avenue for early intervention in bipolar disorder, which focuses on the damaging effects of childhood trauma.^3^ We examine the nature, specificity and effects of these harmful events in bipolar disorder, outline how the two may be mechanistically connected and argue how interventions could ameliorate the onset or attenuate a more aggressive course of bipolar disorder. We, like many, adopt the classical view, whereby bipolar disorder is defined by the onset of first manic/hypomanic episode and largely occurs from late adolescence onward, as opposed to childhood onset. This means that we focus on childhood trauma events or affective instability that predate or co-occur with bipolar disorder onset from late adolescence onward.

## Targeting emotional abuse in bipolar disorder

Children commonly experience trauma, such as physical, sexual and emotional abuse, and this exposure increases their future risk of developing several psychiatric disorders, including bipolar disorder. To offer tailored early intervention for bipolar disorder, one question is whether a specific type of childhood trauma is particularly important in people with bipolar disorder, and in what respect. The emerging picture here is that compared with other trauma subtypes, emotional abuse is greater among people with bipolar disorder compared with controls, and is the trauma subtype most strongly associated with a complex bipolar disorder illness course (i.e. earlier onset, rapid cycling, suicide attempts, increased mood episodes, comorbidity, psychotic features).^4^ The underlying biological mechanisms by which childhood trauma, and particularly emotional abuse, lead to an increased risk of developing bipolar disorder and a worsened bipolar disorder prognosis are far from resolved. Exposure to childhood trauma is associated with long-term changes in the hypothalamic-pituitary-adrenal axis response,^5^ such as increased cortisol reactivity. Thus, individuals exposed to childhood trauma may have heightened responses to subsequent life stressors, rendering them vulnerable to developing mood disorders such as bipolar disorder.^6^

The evidence suggest emotional abuse may be considered a disease modifier (i.e. explaining onset and course of the disorder), and could represent a therapeutic target for early intervention. Taken together, research suggests that, compared with other traumas, there may be a stronger association between emotional abuse and bipolar disorder. One possible explanation for this is that sexual and physical abuse will invariably involve emotional abuse, either predating it or being part of it. Thus, targeting the deleterious effects of emotional abuse in the first instance may be a fruitful first step in developing trauma-focused early interventions for bipolar disorder.

## Affective instability as a potential psychological target

There is now a substantial body of evidence demonstrating that childhood trauma, and emotional abuse specifically, may be more strongly associated with high levels of affective instability.^7^ Evidence indicates affective instability or similar difficulties, such as affective lability or emotional dysregulation, appear early and before the onset of bipolar disorder, and are connected to emotional abuse. Compared with controls, individuals with bipolar disorder report higher rates of affective instability and, more crucially, individuals with bipolar disorder who report childhood trauma also endorse higher rates of affective instability compared with those without trauma histories. Affective instability in bipolar disorder is a potential determinant of a worsened clinical trajectory for bipolar disorder. Path analyses of independent data-sets indicate that affective instability mediates the relationship between childhood trauma and several clinical features of bipolar disorder, such as suicide attempts, mixed episodes, anxiety disorders, earlier age of onset, and number of depressive and manic episodes.^8^ Taken together, this research suggests that affective instability is a worthwhile therapeutic target for bipolar disorder and, because of its transdiagnostic nature, it would likely fit well within prevention frameworks targeting early clinical stages of bipolar disorder. There is ongoing debate about the nosological status and boundaries between bipolar disorder and borderline personality disorder (BPD). Given that childhood trauma is common among both disorders, and affective instability is also a core feature of BPD,^9^ targeting of affective instability may also be part of early interventions for BPD.

## Interventions to target emotional abuse and affective instability

Research shows that both emotional abuse and affective instability are prevalent, correlated and potential drivers to the deleterious outcomes found in bipolar disorder. Thus, we argue that both emotional abuse and affective instability should be: 1) routinely assessed by clinicians, and 2) key therapeutic targets for future interventions (e.g. secondary prevention). In terms of the assessment of emotional abuse and affective instability, there are brief, validated tools that could easily be implemented into routine clinical practice to assess for both. However, one limiting step for this to be seen as acceptable and clinically necessary is the action that might ensue. Presently, it remains unclear how to best target emotional abuse and/or affective instability within people at risk or those who already have a bipolar disorder diagnosis. We offer two key suggestions to help drive this area of research forward: interventions that target emotional abuse (mainly based on the post-traumatic stress disorder (PTSD) literature) and interventions that target emotional-abuse-related affective instability directly (mainly based on the BPD literature).

### Interventions targeting emotional abuse

Current psychological interventions (e.g. family-focused therapy, mindfulness-based cognitive therapy, psychoeducation) do not focus on the psychological effects of childhood trauma, and specifically emotional abuse. One potential treatment avenue would be to explore whether established trauma-focused therapies for PTSD, such as trauma-focused cognitive–behavioural therapy and eye-movement desensitisation reprocessing (EMDR), may also be effective in people with bipolar disorder.^10^ Despite the importance of childhood trauma in bipolar disorder, there are very few studies assessing the efficacy of these trauma-focused therapies in abuse victims at risk of bipolar disorder. To date, there is one pilot study assessing the effects of EMDR versus treatment as usual in patients with bipolar disorder and trauma histories. Promisingly, results showed that EMDR was more effective than treatment as usual in the reduction of depressive, hypomanic and trauma-related symptoms.^11^ There is also one other registered randomised controlled trial currently underway.^12^

Presently, researchers and clinicians in bipolar disorder are heavily reliant upon the PTSD literature to extrapolate inferences about whether these trauma-focused interventions could be useful in bipolar disorder. This is concerning for two reasons: first, because the PTSD literature tends to focus on other forms of trauma (e.g. road-traffic accidents), rather than emotional abuse specifically; second, the traumas assessed in PTSD tend to be single-episode traumas, whereas patients with bipolar disorder have more complex and multiple trauma histories, as well as a serious mood disorder. Hence, it is not known whether these kinds of treatments may be suited and effective for those at risk of or diagnosed with bipolar disorder.

### Interventions targeting affective instability

Interventions targeting affective instability have not been well tested, with this being especially the case (surprisingly) in people with bipolar disorder. Emotional skills training is a short-term intervention that targets better regulation of affect as a key outcome. How far this can be extended to people at risk of bipolar disorder or those with established illness is unknown. Affect regulation is one key part of group-based psychoeducation programmes for bipolar disorder and BPD; namely, dialectical behaviour therapy (DBT) and mindfulness-based cognitive treatment (MBCT).

Although limited, there is growing evidence suggesting that DBT can be successfully adapted to help treat bipolar disorder,^13^ and that DBT may also be effective at targeting affective instability in bipolar disorder specifically. However, further interventional studies (e.g. randomised controlled trials) are needed, particularly in bipolar disorder with a history of emotional abuse. Similarly, another well-established treatment that may hold promise for targeting affective instability in bipolar disorder is MBCT. Reviews suggest that MBCT may be effective for bipolar disorder, with a recent meta-analysis (*N* = 10 studies) reporting that MBCT may help to improve emotional regulation in bipolar disorder from pre- to post-treatment.^14^ To date, there are three trials, and further evidence is needed before we can conclude whether MBCT is effective at targeting affective instability in bipolar disorder or those at risk, who also have a history of emotional abuse.

## Conclusions

Childhood trauma plays a pervasive role in the development and course of bipolar disorder and, in line with recent research, it appears that the effects of childhood trauma and affective instability both pose as suitable therapeutic targets for future bipolar disorder early interventions. Currently, early interventions for bipolar disorder are lacking, particularly ones that are tailored to the needs of those with a history of childhood trauma, and emotional abuse specifically. By failing to address the needs of this clinical population, it is likely that patients with bipolar disorder will continue to experience high rates of relapse and deleterious, if not life-threatening, consequences (e.g. suicide attempts). We recommend that the assessment of childhood trauma needs to be embedded in routine clinical practice to help aid clinical decision-making around treatment plans. We also propose that further research and clinical input is warranted to develop new evidence-based interventions that target the devastating effects of emotional abuse, either via targeting emotional abuse directly (via trauma-focused therapies) or by targeting affective instability (via established emotional regulation-based therapies).
